# The effects of plant extracts on lipid metabolism of chickens — A review

**DOI:** 10.5713/ab.22.0272

**Published:** 2022-11-14

**Authors:** Xuedong Ding, Ilias Giannenas, Ioannis Skoufos, Jing Wang, Weiyun Zhu

**Affiliations:** 1 National Center for International Research on Animal Gut Nutrition, Jiangsu Key Laboratory of Gastrointestinal Nutrition and Animal Health, Laboratory of Gastrointestinal Microbiology National Experimental Teaching Demonstration Center of Animal Science, College of Animal Science and Technology, Nanjing Agricultural University, Nanjing 210095, China; 2 Department of Veterinary Medicine, Aristotle University of Thessaloniki, Campus, 54124, Thessaloniki, Greece; 3 Department of Agriculture, University of Ioannina, Arta, 47100, Greece

**Keywords:** Antioxidant Capacity, DNA Methylation, Hormones, Intestinal Microbiota, Lipid Metabolism, Plant Extracts

## Abstract

The fat deposition is an important factor affecting chicken meat quality, which is closely related to lipid metabolism of chickens. Therefore, it is important to regulate the lipid metabolism of chickens to improve the chicken meat quality. Plant extracts have special regulatory effects on animal’s growth and health and have been widely used in chicken breeding. Some plant extracts have been reported to have functions of changing the fatty acid composition, reducing abdominal fat percentage, and enhancing the intramuscular fat content of chickens by improving the antioxidant capacity, regulating the expression of genes, enzymes, and signaling pathways related to lipid metabolism, modulating intestinal microbiota, affecting hormones level, and regulating DNA methylation. This paper reviewed the application and mechanism of plant extracts on regulating lipid metabolism of chickens to provide a reference for the further application of plant extracts in chicken breeding.

## INTRODUCTION

Plant extracts consist of biologically active components which are extracted and concentrated from plants by physical, chemical, or biological methods without changing their effective structures. These active components are mainly biological micromolecules and macromolecules in plants, including polyphenols, flavonoids, saponins, alkaloids, etc. Most of them are secondary metabolites in the process of plant growth and development, and often have some special functional groups. These non-nutritive organic active components have special regulatory effects on the animal growth and health because of their anti-diabetes, anti-oxidant, anti-cancer, and anti-obesity effects [1]. Therefore, plant extracts have been utilized as feed additives in animal production. In recent years, plant extracts have been widely used in chicken breeding, which can improve productive performance, modulate intestinal microbiota, enhance immunity, and improve antioxidant capacity [2]. Plant extracts have the potential to be used as a substitute for antibiotics to meet the needs of food safety and improve the quality of animal products. In this review, we focus on the effects of plant extracts on fat metabolism of chickens because fat metabolism has an important influence on meat quality.

The fat distribution characteristics of chickens often vary with the growth rate and the breed. In the slow-growing chicken breeds, chickens have more muscle and subcutaneous fat, and the fat distribution is more even [3]. The total fat content in fast-growing chickens is greater than that in slow-growing chickens, and the fat is mainly distributed in the abdomen and on the visceral surfaces. The compositions of monounsaturated fatty acids (MUFA) were lower, and the compositions of polyunsaturated fatty acids (PUFA) were higher in slow-growing than fast-growing chickens [4]. The low intramuscular fat usually results in a poor meat quality [5]. Over the past decades, the growth and development rate of chicken has been greatly improved through continuous genetic selection. However, the excessive abdominal fat deposition results from the accelerated growth rate have been a significant challenge in obtaining high quality of chicken meat. Choct et al [6] found that modern broiler strains contain 15% to 20% carcass fat, of which more than 85% fat is not required by the physiology. Excessive fat deposition in chickens affects the feed utilization rate, and reduces the carcass quality of chicken meat, resulting in the environmental pollution and the loss of economic benefits. Therefore, it is important to control fat metabolism and reduce the excessive abdominal fat deposition. Researchers have made remarkable progress in reducing the abdominal fat percentage of chickens by means of nutrition and heredity [7,8]. However, the reduction of the intramuscular fat content accompanied with the decreased abdominal fat percentage adversely affects the chicken meat quality. Some studies show that plant extracts can regulate fat metabolism of chickens, change the fatty acid composition, reduce abdominal fat percentage, and enhance the intramuscular fat [9–11]. This paper reviewed the application and mechanism of plant extracts on regulating lipid metabolism of chickens in recent years to provide a reference for the further application of plant extracts in chicken breeding and production.

## THE PLANT EXTRACT ADDITIVES USED IN CHICKENS PRODUCTION

A plant extract usually contains different active components depending on various extraction methods [12]. At present, the commonly used extraction processes include solvent extraction, steam distillation, fluid extraction, biological enzyme extraction, and bionic extraction [13]. In recent years, a lot of plant extracts have been used in chicken production because of the prohibition of antibiotics in livestock production. The sources and active components of plant extracts commonly used in chicken production are shown in Table 1. The active components in plant extracts and their by-products including alfalfa, tea, grape seeds, bamboo leaf, mountain celery, soybean, sea buckthorn, citrus grandis peel, and orange peel, have significant effects on the blood lipid level, muscular fat content, muscular fatty acid composition, and the gene expression related to lipid metabolism.

## PLANT EXTRACTS AFFECT THE FATTY ACID COMPOSITION AND IMPROVE THE ANTIOXIDANT CAPACITY OF CHICKENS

Body fat deposition of chickens mainly exists in subcutaneous tissues, abdomen, and intramuscularly, and the intramuscular fat only accounts for a small part of body fat deposition (less than 1%), while subcutaneous fat and abdominal fat are main deposition forms [31]. The content of intramuscular fat in thigh muscle is greater than that in breast muscle [32]. But the fatty acid composition is relatively stable, and most of fatty acids are unsaturated fatty acids (UFA) accounting for more than 60% of total fatty acids, including palmitic acid, oleic acid, and linolenic acid, etc. [33]. In chicken thigh and breast muscle, the fatty acid composition of intramuscular fat does not have a significant difference among different breeds, and the fatty acids are mainly MUFA and PUFA [4]. The UFA not only improve the flavor of chicken meat, but also are a kind of indispensable nutrients for human body. The UFA can eliminate free radicals, increase blood low-density-lipoprotein (LDL), reduce blood cholesterol, and improve blood circulation [34]. However, UFA are easy to be spontaneously oxidized. Therefore, the prevention of UFA spontaneous oxidation is important for improving the quality of chicken meat.

Studies have shown that plant extract additives can improve the antioxidant capacities and reduce the lipid peroxidation, which may affect the contents of serum triglyceride (TG) and LDL, influence fat deposition and fatty acid composition of chickens [8–11]. According to recent studies, the plant extracts with antioxidant functions include alfalfa flavonoids [16], mountain celery [35], soy isoflavone [36], hesperidin [37], Eucommia ulmoides leaf [38], grape seeds extract [20], bamboo leaf extract [22], etc. The antioxidant capabilities of plant extracts include increasing the activity of superoxide dismutase (SOD), catalase (CAT), and glutathione peroxidase (GSH-Px) in serum and tissues of chickens, increasing total antioxidant capacity (T-AOC) and decreasing the concentration of malondialdehyde and lipid oxidation of chicken meat during cold storage [22]. The mechanism may relate to regulating the expression of some antioxidant genes and their signaling pathways. Gene analysis showed that mountain celery (7.5 g/kg) could reduce a lipid peroxidation level by up-regulating the expression of *SOD1* and *CAT* in broiler liver [35]. Genistein (40 mg/kg) enhanced the antioxidant activity of chicken by up-regulating the expression of genes (*SOD3*, metallothionein 1, and 4), enhanced the activities of T-AOC and T-SOD in chicken liver [39]. Nuclear factor E2-related factor 2 (Nrf2) and peroxisome proliferator-activated receptor α (PPARα) are the important signaling pathways related to antioxidant capacity. Chlorogenic acid-enriched extract (CGAE) from Eucommia ulmoides leaf (1 g/kg) increased the expression of *Nrf2*, *SOD*, and *CAT* in heat-stressed chicken breast muscle, and improved the oxidation stability and changed the fatty acid composition of heat-stressed chicken breast muscle by activating *Nrf2* [38]. Bamboo leaf extract (BLE, 2.0 to 3.0 g/kg) reduced the lipid oxidation and increased the antioxidant capacity of chicken breast muscle by activating *Nrf2*. The BLE significantly up-regulated the mRNA expression of *Nrf2*, *GSH-Px*, heme oxygenase 1 (*HO-1*), and glutathione S-transferases (*GSTs*) gene in chicken breast muscle [23]. Hesperdin and naringin (0.75 g/kg) significantly increased the expression of *PPARα*, acyl coenzyme A oxidase 1 (*ACOX1*), and glutathione disulfide reductase (*GSR*) in broiler liver, promoted the fatty acid β-oxidation in liver, changed fatty acid composition in breast muscle and reduced abdominal fat of broiler chickens [9]. Table 2 lists some plant extracts which have significant effects on fatty acid composition, content, and their ratio in chickens.

## PLANT EXTRACTS REGULATE THE LIPID METABOLISM AND FAT DEPOSITION IN CHICKENS

### Plant extracts regulate the expression of genes, enzymes, and signaling pathways related to lipid metabolism and fat deposition in chickens

Liver is chicken’s main lipid synthesis organ where fatty acids from various sources were esterified to form TG, phospholipids, and cholesterol esters, among which TG are main raw materials for abdominal, subcutaneous, and muscle fat synthesis. The TG synthesized by liver were carried by the very low-density lipoprotein (VLDL) through the blood circulation and reached various tissues for the utilization or storage. The distribution difference between abdominal and intramuscular fat in chickens is influenced by some complicated factors. i) Lipoprotein lipase (LPL) is the most important factor affecting chicken fat deposition. The LPL catalyzes the decomposition of chylomicron and VLDL into fatty acids and monoglycerides. LPL-catalyzed lipoprotein hydrolysis is the speed-limiting step for plasma lipoprotein to enter peripheral tissues [45]. The missense mutation of the 377th base in the carboxyl terminal domain of *LPL* gene can improve the binding ability of LPL and VLDL, thereby enhancing TG metabolism in VLDL and promoting tissue fat deposition [46]. In broilers, the abdominal fat deposition is faster than that of laying hens due to the higher LPL activity [47]. The fat accumulation in broilers can be reduced by injecting LPL monoclonal antibody to inhibit the LPL activity in adipose tissue [48]. ii) Adipocyte fatty acid binding protein (A-FABP) is another important factor affecting chicken fat deposition because the mRNA expression of *A-FABP* is positively correlated with the intramuscular fat content [49]. Three genotypes of the A-FABP gene first exon, AA, AB, and BB were examined by polymerase chain reaction-single-strand conformation polymorphism. The intramuscular fat content with the AA genotype was significantly higher than that with the AB genotype in the Baicheng-You Chickens [50], three-Yellow Chickens and Hetian-Black Chickens [51]. iii) According to the research of Fu et al [52], five transcriptional regulatory factors including CCAAT/enhancer binding protein α and β (CEBPα, β), PPARα, PPARγ, sterol-regulatory element binding proteins (SREBP-1) influenced the fat deposition of Beijing-You chickens. CEBPα and PPARγ are important factors because the expression of CEBPα and PPARγ in abdominal fat was significantly greater than that in breast and thigh muscle, while the expression of PPARα in breast and thigh muscle was significantly greater than that in abdominal fat. The fat content in breast muscle of Beijing-You Chickens was significantly lower (p<0.05) than that in thigh muscle, and the fat deposition in breast and thigh muscle was two different expression patterns. Gene chip analysis showed that the expression levels of *PPARγ*, *LPL*, *FABP4*, thyroid hormone responsive, retinoid-binding protein 7, *FABP3*, and *LDL* receptor in breast muscle were lower than those in thigh muscle, while the expression levels of retinoid X receptor alpha and *CEBPβ* in breast muscle were significantly higher than those in thigh muscle, which suggested that *PPARγ* and its downstream genes played an important regulatory role in intramuscular fat deposition [53].

Studies have shown that plant extracts affected chicken fat deposition by regulating the level of TG, total cholesterol (TC), LDL, and VLDL in serum and the expression of genes, enzymes, and signaling pathways related to fat synthesis, decomposition, and transportation in liver, muscle, and abdominal fat [2]. Among these plant extracts, soy isoflavone, flavonoids of sea buckthorn fruits (FSBF), alfalfa flavonoids, rutin, quercetin, curcumin, tea polyphenols, and EGCG can significantly reduce the contents of TG, TC, and LDL in serum and abdominal fat percentage in broiler chickens. Some plant extracts, such as quercetin and rutin can decrease fat deposition by regulating the expression of genes related to fat synthesis and decomposition in chickens. High concentration rutin (0.5 to 1 g/kg) can significantly increase carnitine palmitoyl transferase 1 (CPT1), decrease acetyl CoA carboxylase (ACC) and fatty acid synthase (FAS) in liver [54]. Quercetin inhibited the adipogenesis and fat deposition by regulating cAMP signaling pathway, when chicken’s adipocyte cells were cultured with quercetin, the contents of ACC, FAS, LPL, TG, and phosphodiesterase (PDE) were significantly decreased, while the contents of cyclic adenosine monophosphate and protein kinase A were significantly increased [55]. Quercetin (0.6 g/kg) significantly increased the mRNA expression of *PPARα*/AMPK signaling pathway and accelerated lipolysis and fatty-acid oxidation; while decreasing the mRNA expression of *ACC*, 3-hydroxy-3-methyl glutaryl coenzyme A reductase (*HMGR*), *SREBP1*, and *PPARγ* to attenuate fat accumulation [56]. The analysis based on gene ontology and Kyoto encyclopedia of genes and genomes database showed that quercetin also reduced abdominal fat percentage by influencing the ileum fat digestion and absorption, glycerol phospholipid metabolism, AMPK signaling pathway, fatty acid degradation, and cholesterol metabolism, and resulted in the decrease of abdominal fat. In addition, the percentage of fat content in thigh muscle tended to increase according to the increased quercetin addition dose [57].

Tea polyphenols have an excellent lipid-lowering effect. Green tea polyphenols (GTP, 80–160 mg/kg) reduced the blood lipid level and obesity of broiler chickens by inhibiting fatty acid synthesis and stimulating lipolysis. The GTP enhanced the phosphorylation level of AMPKα and acetyl-coenzyme A carboxylase alpha [58], down-regulated the expression level of liver lipid anabolic genes (malic enzyme), up-regulated the expression of genes and transcription factors related to lipid oxidation in liver (*CPT-I*, *ACOX1*, and *PPARα*), and up-regulated adipose triglyceride lipase (*ATGL*) in abdominal fat and LPL in skeletal muscle [18]. Epigallocatechin gallate (EGCG) is an important component of tea polyphenols, two weeks of 40 to 80 mg/kg EGCG diet significantly reduced the abdominal fat deposition of the broilers, significantly down-regulated fatty acid synthesis and gene expression related to fat deposition, up-regulated gene expression related to fatty acid β-oxidation and lipolysis, significantly decreased the activities of fatty acid synthetases (FAS and ACC) and increased the activity of CPT-1 in liver [59].

Soy isoflavone is a flavonoid extracted from soybean. Adding 30 mg/kg soybean isoflavone to a broiler diet significantly increased the expression of fatty acid desaturase (*FADS*) 1 and 2, enhanced the expression elongase of very long chain fatty acids (*ELOVL*) 2 and 5 genes, and decreased the percentage of abdominal fat in broilers (p<0.05) [60]. Genistein is an active component in soybean isoflavones, and adding 40 to 400 mg/kg genistein to a broiler diet increased the activities of LPL and liver lipase, and down-regulated the expression of genes related to lipid synthesis including *SREBP-1c* and *FAS*, resulted in the decreased accumulation of lipid in liver [14]. Genistein treatment also enhanced fatty acid β-oxidation and up-regulated *PPARδ* expression in chicken liver. ChIP-qPCR analysis showed that genistein supplementation in hen’s diet changed the lipid metabolism of offspring chicks through the epigenetic modification, induced histone H3-K36 trimerization in *PPARδ* gene promoter region through SUPT6 interacting protein methyltransferase, induced histone H4-K12 acetylation in PPARδ promoter through MYST histone acetyltransferase 2, and activated the PPAR signaling pathway in chicken liver [39].

Alfalfa flavonoids (15 mg/kg) up-regulated LPL, ATGL, PPARγ, and down-regulated FAS expression in fat and liver tissues of female Arbor Acre broilers [16], decreased the fat deposition of female Chongren Chickens at the late growth stage. Upon the treatment, the thickness of subcutaneous fat was 26.22% and the width of intramuscular fat was 20.53% lower than those of the control group, and the percentage of abdominal fat was 11.66% and the content of thigh muscle fat was 9.35% lower than those of the control group [61]. Bamboo leaf extract (2.0 to 3.0 g/kg) promoted the conversion of low-density lipoprotein cholesterol (LDL-c) into bile acid by up-regulating the expression of *LDL* receptor and cholesterol 7-alpha-hydroxylase (*CYP7A1*) mRNA, and down-regulating the expression of hydroxy-3-methylglutaryl coenzyme A reductase (*HMGR*) to decrease cholesterol synthesis and to improve cholesterol metabolism, and resulted in the decreased content of TG and LDL-c in serum and abdominal fat deposition [22]. Lycopene (400 mg/kg) reduced abdominal fat deposition and blood lipid level in broiler chickens, probably through the regulation of lipid metabolism by activating AMPK signaling pathway [10]. Curcumin (2,000 mg/kg) reduced liver and plasma lipid level and influenced the expression level of genes related to lipogenesis and lipolysis including *ACC*, *FAS*, *SREBP-1c*, ATP-citRate synthase, *PPARα*, and *CPT-I* [11], and decreased abdominal fat deposition.

### Plant extracts through DNA methylation regulate the expression of genes and signaling pathways related to lipid metabolism in chickens

The most important function of DNA methylation is to regulate gene transcription and expression activity. Studies showed that DNA methylation and demethylation affected the proliferation and differentiation of adipocytes, and participated in regulating the expression of transcription factors, transcription cofactor coding genes and adipose tissue-specific genes during adipocyte differentiation, thus influencing the growth and development of the fat deposition in abdomen, subcutis, and muscle [62]. Sun et al [63] discovered that for abdominal adipose tissue, the methylation degree of *PPARγ* gene promoter region in low fat chickens was significantly greater (p<0.0001) than that in high fat chickens, and its mRNA expression in low fat chickens was lower than that in high fat chickens. Therefore, regulating the DNA methylation process which is related to lipid metabolism gene could regulate fat deposition in chickens. Betaine, also known as trimethylglycine, is an important methyl donor and can regulate DNA methylation process. Betaine promoted fat metabolism, reduced abdominal fat percentage, and significantly decreased fat content in liver of laying hens [30,64]. The main mechanisms of betaine regulating the broiler fat deposition include i) reducing the content of nicotinamide adenine dinucleotide phosphate, a hydrogen donor needed for fatty acid synthesis and carbon chain extension [65], ii) promoting the synthesis of carnitine and the β-oxidation of fatty acids in liver [30], iii) affecting the activities of hormone sensitive lipase (HSL) and LPL in abdominal fat [66]. Studies showed that adding betaine to the diet reduced the concentration of LDL, TG, and TC in serum of broilers, and increased the concentration of nonestesterified fatty acids. Betaine also increased the mRNA expression of *PPARα*, *CPT-1*, and 3-hydroxyacylcoenzyme a dehydrogenase (*HADH*), and decreased the mRNA expression of *FAS* and *HMGR* in liver, which decreased fatty acid synthesis and increased β-oxidation, and decreased abdominal fat of broiler chickens [30]. Heat stress increased the fat deposition in the abdomen, muscle, and subcutis of broiler chickens, while supplementing 0.1% betaine significantly reduced the concentration of TG, free fatty acids, LDL, and HDL in serum of broilers under heat stress, and alleviated the fat deposition in abdomen, subcutis, and muscle [67].

Xing et al [68] discovered that the mRNA levels of *LPL*, *FAS*, and *A-FBP* in broilers abdominal fat were significantly decreased when 0.1% betaine was added to the broiler diet. The CpG methylation analysis of LPL promoter region showed that CpG methylation degree at the 1st, 6th, 7th, 8th, and 10th to 50th positions was lower, while the methylation degree at the 2nd, 5th, and 9th positions was higher. Betaine could also regulate the CpG methylation level of the gene promoter of SREBP2 and CYP7A1 in chicken liver and resulted in the decreased cholesterol biosynthetic enzymes (SREBP2 and HMGR) activities and the increased CYP7A1 activities, thereby reducing cholesterol synthesis and promoting cholesterol decomposition. Meanwhile, the mRNA and protein expression of *LDL* receptor in liver was increased, and the mRNA abundance of acyl-CoA:cholesterol acyltransferase 1, which mediates cholesterol esterification, was decreased significantly, and this epigenetic regulation of *SREBP2* and *CYP7A1* genes by betaine decreased the liver cholesterol deposition of offspring chickens [64]. In addition, the intraperitoneal injection of betaine (2.5 mg/egg) into eggs significantly increased the mRNA abundance of *SREBP1* and ATP binding box subfamily a member 1 and regulated the cholesterol reverse transport in the liver of newly hatched chicks. At the same time, the protein content of DNMT1 and adenosine homocysteinase 1 was increased in liver, which was related to the enhanced methylation of genomic DNA and the decreased inhibition of histone H3 lysine 27 trimethylation gene [69].

### Plant extracts regulate lipid metabolism and fat deposition by affecting hormones level in chickens

Fat deposition is a complex process which is related to energy regulation and fat metabolism. Studies showed that some hormones influenced lipid metabolism in chickens. For example, follicle-stimulating hormone (FSH) regulated chicken fat deposition by manipulating the genes related to lipid metabolism including *FAS*, *LPL*, diacylgycerol acyltransferase 2, *A-FABP*, and *PPARγ* in abdominal fat and breast muscle. As a result, the accumulation of abdominal fat and muscle fat was significantly increased when the chickens were stimulated by FSH receptor [70]. After the castration, the testosterone level of chickens was decreased, which could up-regulate lipid synthesis gene (apolipoprotein A1, stearoyl-CoA desaturase, *FABP7*, retinoid X receptor-gamma, and fatty acid desaturase 2) in abdominal fat through a PPAR pathway, and resulted in a significant increase of abdominal fat content [71]. Huang et al [72] and coworkers showed that brain natriuretic peptide (BNP) and its receptor (NPR1) were involved in regulating chicken abdominal fat metabolism. *BNP* and *NPR1* mRNA were significantly up-regulated in chickens with high abdominal fat because BNP stimulated the proliferation, differentiation and lipid decomposition of pre-adipocytes by NPR1 and the key genes of glycerol lipid metabolism. The concentrations of insulin and adiponectin were closely related to the fat deposition and distribution. Insulin is an effective lipogenic hormone which induces lipogenesis and lipid accumulation in chicken embryos [73]. Adiponectin is a protein hormone secreted by adipocytes and reduces C/EBPα in chicken preadipocyte [74]. The intramuscular fat content was positively correlated with insulin and leptin, and negatively correlated with adiponectin [28]. Studies have showed that some plant extracts have a hormone regulating effect. The dietary supplementation with genistein (50 to 150 mg/kg) improved the secretion of adiponectin or indirectly up-regulated Erβ through the paracrine effect of adiponectin to decrease the fat accumulation in broilers. This process might relate to the activation of AMPK-sirt1/PGC-1α signaling pathway [75]. Adding FSBF (5 to 10 g/kg) to the broiler diet increased the content of insulin in serum and intramuscular fat content in breast and thigh muscle and decreased the content of adiponectin in serum and the percentage of abdominal fat. Quercetin (0.4 to 0.6 g/kg) significantly increased the content of leptin and adiponectin in serum and decreased the percentage of abdominal fat in broilers [57]. The glucocorticoid receptor (GR), known as a regulator of the genes that encoded fatty acid and TG synthetase, could regulate fat metabolism and promoted fat deposition in broilers’ muscle and liver [76]. Steroid acute regulatory protein (STAR) is a rate-limiting protein which transported cholesterol to the mitochondrial intima. Betaine-supplemented (5%) in diet significantly increased the mRNA expression of *STAR* and *GR* in adrenal gland [77]. Betaine could also affect the expression of liver lipid synthesis and regulate the expression of lipid transport-related genes by modifying the methylation. Chromatin immunoprecipitation analysis showed that betaine increased the binding of GR to *SREBP1* and *apoB* gene promoters [78]. Thyroid hormone regulated the energy metabolism in the body, and the decreased circulating thyroid hormone level is a protective mechanism to reserve energy. Nuciferine influenced body weight in broilers by varying the content of thyroid hormone in plasma, and regulated lipid metabolism to decrease fat deposition. Adding nuciferine to a broiler diet decreased the plasma concentration of thyroxine and free thyroxine and increased the TC oxidation, the concentrations of TG and glucagon in plasma [79]. Since the fat metabolism of chickens is affected by many hormones whose level can be influenced by plant extracts, the mechanism of plant extracts on fat metabolism needs to be further explored.

### Plant extracts regulate lipid metabolism and fat deposition by affecting intestinal microbiota of chickens

Intestinal microbiota is highly adaptable to the micro-environment of different intestinal segments which has different diversity, composition, and potential functions in different parts of intestine. Moreover, host inheritance also shapes intestinal microbiota which plays an important role in animal health and growth performance. 16S rRNA gene sequence analysis showed that the main microbes in chicken intestine were Firmicutes, Bacteroides, Proteobacteria, and Actinomycetes. Firmicutes was a dominant bacterial phylum in duodenum, jejunum, ileum, and colon (>60%), while Bacteroides was a dominant bacterial phylum in cecum (>50%). At the genus level, the major microbial genera in intestine were *Lactobacillus*, *Enterococcus*, *Bacteroides*, and *Corynebacterium*. *Lactobacillus* was a predominant genus in duodenum, jejunum, and ileum (>35%), but was rarely present in cecum, and *Bacteroides* was the most dominant group in cecum (about 40%) [80]. Many studies showed that plant extracts influenced the composition and abundance of intestinal microbiota including the inhibition of harmful bacteria and the promotion of beneficial bacteria [81–84]. Laminarin-rich extract (300 ppm) increased the abundance of *Bifidobacterium* in broilers cecum [81]. Saccharide-enriched *Acanthopanax senticosus* extract (1 g/kg) increased the abundance of *Lactobacillius* and decreased the abundance of *Escherichia coli* and *Salmonella* in ileum [82]. Rosemary volatile oil (5.7 to 11.5 g/kg) significantly reduced the abundance of cecal *Escherichia coli* in broiler chickens [83]. Genome-wide sequencing and 16S rRNA gene sequencing results showed that the cecal *Mucispirillum schaedleri* abundance was negatively correlated with the chicken abdominal fat level, while the cecal *Methanobacteriaceae* abundance was positively correlated with the chicken fat deposition [84]. Firmicutes and Bacteroides can influence fat deposition and the greater ratio of Firmicutes and Bacteroides resulted in the greater fat storage [85]. *Clostridium butyricum* increased intramuscular fat content by promoting breast muscle lipogenesis [86] and reduced the breast muscle fatty acid oxidation [87]. Adding *Clostridium butyricum* to the broiler diet increased serum insulin level and LPL activity, up-regulated *FAS*, malic enzyme (*ME*), *ACC* mRNA expression in liver and *LPL* mRNA expression in breast muscle, decreased HSL activity and the mRNA levels of CPT 1, 2, and long chain acyl coenzyme A dehydrogenase in muscle, reduced muscle fatty acid oxidation, and promoted muscle fat deposition through modulating cecal microbiota [86,87]. Adding *Lactobacillus acidophilus* to the broiler diet reduced the levels of TC, TG, and VLDL in serum, and decreased the abdominal fat level [88]. Adding *Enterococcus faecium* to a broiler diet reduced the leptin content in serum, increased mRNA level of *FAS*, *ME*, and *ACC* in liver [86]. These results suggested that intestinal microbiota influenced the fat synthesis and fat deposition, but the mechanism remains unknown.

Short-chain fatty acids (SCFAs) including acetic acid, pro pionic acid, and butyric acid, etc. are produced by intestinal microbiota and influence the host metabolism. *Bacteroidetes* mainly produces acetic acid and propionic acid, and butyric acid is the main metabolic end product of Firmicutes. Probiotics treatments in diet changed the cecal microbiota diversity, increased the content of acetic acid and butyric acid, which induced intestinal GLP-1 secretion through AMPK pathway, while GLP-1 further reduced liver fat synthesis by activating AMPK/ACC pathway in chickens, which resulted in a low TG/TCH level and a low abdominal fat percentage [89]. Studies showed that butyrate and propionate regulated the release of intestinal hormones in mice, which inhibited food intake and prevented obesity caused by diet [90]. Adding sodium propionate into the diet decreased the fat deposition of broilers by reducing the food intake [91]. Adding a low-dose of butyrate to the broiler diet down-regulated fatty acid receptor-mediated lipogenesis genes and decreased fat deposition in liver and abdomen, while a high-dose butyrate diet promoted fat deposition in broiler chickens [92]. These results indicated that intestinal microbiota influences the chicken fat deposition by affecting the production of SCFAs. Therefore, it is possible to control the chicken fat deposition by changing the composition of intestinal microbiota. During simulated fecal fermentation *in vitro*, ginger extract promoted the growth of *Bifidobacterium* and *Enterococc*, elevated the levels of SCFAs [93]. *Macleayacordata* extract (400 mg/kg) in a broiler diet promoted the abundance of *Lactobacillus* and *Clostridium cluster XIVa* bacteria in the cecum, and increased the concentrations of acetic acid and butyric acid in cecal chyme [94]. Green tea powder (10 g/kg) increased the abundance of *Lactobacillus* in ileum and cecum, increased the content of thigh and breast muscle, enhanced the intramuscular fat content and decreased the abdominal fat content of broiler chickens [95]. When the eucalyptus leaf polyphenols extract (500 mg/kg) was fed to broiler chickens, the muscle fatty acid composition was influenced and the ratio of Firmicutes/Bacteroides in cecum was increased [96]. These results suggested that plant extracts can regulate fat deposition in chickens through intestinal microbiota and SCFAs, but the specific mechanism is still unclear and needs a further investigation.

## CONCLUSION

Although some studies showed the effects of plant extracts on fat metabolism in chickens, the specific mechanism remains unclear. In this paper, some mechanisms of plant extracts regulating fat deposition in chickens were reviewed. However, the active components of plant extracts vary widely, and the extraction process and purity technology influence the biological effects of plant extracts. To have a wide applications of plant extracts in chicken breeding and production, the research direction should focus on exploring the active ingredients and understanding the mechanisms in the future. The metabolic transformation process, tissue distribution, and the residues of plant extracts need a further investigation.

## Figures and Tables

**Table 1:** Commonly used plant extract additives in chicken production

Name	Source and extract method	Major component	Effects in chickens
Soy isoflavone/Genistein [14]	Soybean isoflavones are a kind of secondary metabolites from the growth of soybean, which can be extracted by an organic solvent.	100 g of soybean contains 128 mg of isoflavones, and about 102 mg of isoflavones. It mainly includes genistein, daidzein, and glycitein, among which genistein is the isoflavone with the most content in soybean.	Genistein supplementation inhibited fatty acid synthesis, enhanced β-oxidation, and improved the antioxidant capacity in the liver of chickens.
Quercetin (Rutin) [8]	Quercetin is widely distributed in plants, and can be obtained by ethanol extraction, enzyme or acidic aqueous solution extraction.	Quercetin content is usually less than 10 mg/kg in vegetables, about 40 mg/kg in beans, apples, black vegetables, and broccoli, and more than 100 mg/kg in onions, lettuce, beans, and kale. The quercetin-3-O-rutin (rutin) is one of the main forms, and the onion quercetin-4′-O-glucoside, and quercetin-3,4′-O-glucoside are the most abundant component.	Quercetin supplementation improved growth performance, antioxidant capacity, stability of lipids, and fatty acid composition in breast meat of chickens.
Alfalfa flavonoids [15,16]	Alfalfa flavonoids are mainly obtained from the stems and leaves of alfalfa by an organic solvent extraction and ultrasonic extraction.	The content of flavonoids in alfalfa leaves is the highest (0.86%), followed by stems (0.40%), and flowers (0.26%), including apigenin, luteolin, rutin, tricin, vestitol, sativanone, laricytrine, larivitrin, kaempferol, quercetin, liquritigenin, genistein, and myricetin.	Alfalfa flavonoids supplementation improved average daily gain and breast percentage, meat quality, and antioxidant activity of chickens.
Tea polyphenols [17,18]	Tea polyphenols are the general name of polyphenols in tea. The content of tea polyphenols in green tea is high, accounting for 15%~30%.Tea polyphenols are obtained by a solvent extraction method.	Tea polyphenols include flavones, anthocyanidins, flavonols, anthocyanidins, phenolic acids, and condensed phenolic acids, among which flavanones (mainly catechins) are the most important component, accounting for 60% to 80% of tea polyphenols, and the second important one is flavonoids.	Green tea polyphenols supplementation alleviated obesity and serum lipid levels in chickens by suppressing fatty acid synthesis and stimulating lipolysis.
Grape seeds extract (GSE) [19,20]	GSE is obtained from grape seed by a solvent extraction, a microwave extraction, and an ultrasonic extraction.	Total polyphenol content >40% (including 5% monomer flavan -3- ol and 30% procyanidin).	GSE supplementation improved antioxidant and immunostimulant agent, decreased total cholesterol and low-density lipoprotein in chickens.
Pomegranate by-products (PB) [21]	PB is obtained from pomegranate peel and pomegranate seed by a water extraction, acid-base extraction, a fermentation or a macroporous resin separation.	PB includes polyphenols, flavonoids, anthocyanins, and other phenolic compounds.	PB Supplementation improved meat composition, fatty acid profile, and oxidative stability of chicken meat.
Bamboo leaf extract [22,23]	Bamboo leaf extract is obtained from bamboo leaves by an alcohol extraction.	Bamboo leaf extract includes flavonoids and polyphenols (Flavonoid content in bamboo leaves is 70 mg/g, and polyphenol content in bamboo leaves is 50.42 mg/g).	BLE supplementation improved antioxidant status and cholesterol metabolism, decreased serum triglyceride, low-density lipoprotein cholesterol content, and abdominal fat deposition of broilers.
Curcumin [11,24]	Curcumin is a diketone compound obtained from the rhizomes of some plants in zingiberaceae and araceae by a water extraction, and a solvent extraction.	Curcumin	Curcumin supplementation reduced abdominal fat deposition, decreased the hepatic and plasma lipid profile in chickens.
Hesperidin and naringin [9,25]	Hesperidin is obtained from the peel of mature pericarp and citrus fruits by an acid-base extraction.	Dihydroflavone glycoside	Hesperidin and naringin supplementation beneficially affected fatty acid profiles in the breast meat and fat pad of chickens.
Lycopene [10,26]	Lycopene is obtained from red, yellow or orange vegetables, and fruits, especially tomatoes and carrots by a biological fermentation and a lipophilic organic solvent extraction.	Carotenoid	Lycopene supplementation alleviated abdominal fat deposition and decreased serum lipids levels of chickens.
Flavonoids of seabuckthorn fruits (FSBF) [27,28]	Flavonoids of sea buckthorn are obtained by a reflux extraction and an ultrasonic extraction. The content of flavonoids in seabuckthorn fruit is 3.65 μg/g of fruit juice, 3.54 μg/g of flesh, 4.9 μg/g of peel residue, 1.38 μg/g of seeds, and 8.76 μg/g of seabuckthorn leaves.	Flavonoids in seabuckthorn fruit exist in the form of aglycones, and the main types are isorhamnetin (72%), quercetin (21%), and kaempferol (7%).	FSBF supplementation affected growth performance and fat deposition of chickens by regulating lipometabolism.
Betaine [29,30]	Betaine is obtained from the roots, stems, leaves, and fruits of natural plants, beet, lycium barbarum, and leguminosae by a water extraction and an alcohol extraction. Betaine is an intermediate product of animal metabolism.	Betaine	Betaine supplementation reduced abdominal fat deposition of chickens in a dose-dependent manner.

**Table 2:** Effect of plant extracts on fatty acids composition, content, and ratio in chickens

Name	Effects	Experimental information
Quercetin [8,40]	Quercetin reduced the content of saturated fatty acids (SFA), total polyunsaturated fatty acids (PUFA), palmitic acid, oleic acid, and linoleic acid in breast muscle.	300 (1-day-old) Hubbard strain male broiler were administered with 100, 200, and 300 mg quercetin per kg of feed for 6 weeks.
Genistein [39,41]	Genistein decreased the contents of SFA, total monounsaturated fatty acids (MUFA), and n-3 fatty acids in breast muscle, and increased the contents of PUFA, n-6 PUFA, n-6/n-3 PUFA ratio, and PUFA/SFA ratio, including the myristic acid (C14:0), stearic acid (C18:0), palmitoleic acid (C16:1), oleic acid (C18:1), α-linolenic acid (a-18:3), γ-linolenic acid (r-18:3), and docosahexaenoic acid (c22:6) were decreased and linoleic acid (C18:2). Genistein reduced the contents of long-chain fatty acids, cholesterol, and triglycerides in chicken liver. The contents of n-6 PUFA, total MUFA, and PUFA in liver, including myristic acid (C14:0), eicosapentaenoic acid (C20:5), docosahexaenoic acid (C22:6), palmitic acid (C16:0), heptadecanoic acid (C17:0), and oleic acid were decreased. However, the contents of arachidonic acid (C20:4) and eicosanoic acid (C23:0) were increased.	360 (1-day-old) mixed-sex Arbor Acre broiler chicks were administered with 5 mg genistein per kg of feed for 6 weeks.
Hesperidin and naringin [9]	Hesperidin and naringin decreased the content of oleic acid and increased the contents of linoleic acid and α-linolenic acid. The contents of SFA in breast muscle were decreased (5% to 7%) due to the decrease of caproic acid (C6:0), palmitic acid and stearic acid, and the total PUFA, while the contents of n-6 fatty acids, total PUFA (8% to 10%), and PUFA/SFA ratio were increased due to the increase of linoleic acid. Hesperidin and naringin decreased the content of docosahexaenoic acid in thigh muscle, increased the ratio of n-6/n-3 PUFA and the contents of total PUFA (increased by 8.5% to 11%), n-6 PUFA (increased by 9% to 10%), and PUFA/SFA ratio in abdominal fat.	240 (1-day-old) Ross 308 broiler chickens were administered with 0.75 and 1.5 g naringin per kg of feed for 6 weeks.
Curcumin [42]	Curcumin decreased the contents of SFA, including the lauric acid (C12:0) and stearic acid, and increased the contents of total PUFA and MUFA, including the heptadecanoic acid, heptadecenoic acid (C17:1), behenic acid (C22:0), eicosenoic acid (C20:1), tetradecanoic acid (C24:1), and linoleic acid in breast muscle.	225 (1-day-old) male Cobb 500 strain broiler chickens were administered with 50 mg curcumin per kg of feed for 44 days.
Fermented pomegranate by-products (FPB) [43,44]	FPB increased the contents of MUFA and n-3 PUFA in breast and thigh muscle, and decreased the contents of cholesterol, SFA, and n-6/n-3 PUFA. The contents of stearic acid and SFA in breast muscle were decreased, while the contents of oleic acid and eicosapentaenoic acid were increased. The contents of palmitic acid, stearic acid, SFA, MUFA, oleic acid, eicosapentaenoic acid, and α-linolenic acid in thigh muscle were increased.	320 (1-day-old) Ross 308 male broiler chicks were administered with 5, 10, and 20 mg FPB per kg of feed for 6 weeks.
Flavones of seabuckthorn fruits (FSBF) [28]	FSBF increased the contents of total UFA, MUFA, total PUFA, and the ratio of UFA/SFA in breast and thigh muscle, including the myristic acid (C14:1), palmitoleic acid (C16:1), stearic acid, eicosenoic acid, linoleic acid, α-linolenic acid, eicosadienoic acid (C20:2), dihomo-γ-linolenic acid, docosahexaenoic acid, and eicosadienoic acid.	240 (1-day-old) Arbor Acres male broilers were administered with 5, 10, and 15 mg FSBF per kg of feed for 6 weeks.
Chlorogenic acid-enriched extract (CGAE) [38]	CGAE decreased the contents of stearic acid and SFA in breast muscle and increased the contents of total PUFA and n-6 PUFA, the ratio of PUFA to SFA, including the dihomo-γ-linolenic acid, linoleic acid, linolenic acid, and eicosapentaenoic acid.	400 (28-day-old) male Ross 308 broilers were administered with 0.5 and 1 g CGAE per kg of feed for 14 days.
